# Comparison of different dose accumulation strategies to estimate organ doses after stereotactic magnetic resonance-guided adaptive radiotherapy

**DOI:** 10.1186/s13014-023-02284-7

**Published:** 2023-05-29

**Authors:** Sebastian Regnery, Lukas Leiner, Carolin Buchele, Philipp Hoegen, Elisabetta Sandrini, Thomas Held, Maximilian Deng, Tanja Eichkorn, Carolin Rippke, C. Katharina Renkamp, Laila König, Kristin Lang, Sebastian Adeberg, Jürgen Debus, Sebastian Klüter, Juliane Hörner-Rieber

**Affiliations:** 1 Department of Radiation Oncology, Heidelberg University Hospital, Im Neuenheimer Feld 400, 69120 Heidelberg, Germany; 2 National Center for Radiation Oncology (NCRO), Heidelberg Institute for Radiation Oncology (HIRO), Im Neuenheimer Feld 400, 69120 Heidelberg, Germany; 3 National Center for Tumor diseases (NCT), Heidelberg, Germany; 4 Clinical Cooperation Unit Radiation Oncology, German Cancer Research Center (DKFZ), Heidelberg, Germany

**Keywords:** Stereotactic body Radiotherapy (SABR), Image-guided Radiotherapy (IGRT), MR-guided adaptive radiotherapy, Pulmonary Cancer, Liver Cancer, Deformable image Registration, Dose accumulation

## Abstract

**Introduction:**

Re-irradiation is frequently performed in the era of precision oncology, but previous doses to organs-at-risk (OAR) must be assessed to avoid cumulative overdoses. Stereotactic magnetic resonance-guided online adaptive radiotherapy (SMART) enables highly precise ablation of tumors close to OAR. However, OAR doses may change considerably during adaptive treatment, which complicates potential re-irradiation. We aimed to compare the baseline plan with different dose accumulation techniques to inform re-irradiation.

**Patients & methods:**

We analyzed 18 patients who received SMART to lung or liver tumors inside prospective databases. Cumulative doses were calculated inside the planning target volumes (PTV) and OAR for the adapted plans and theoretical non-adapted plans via (1) cumulative dose volume histograms (DVH sum plan) and (2) deformable image registration (DIR)-based dose accumulation to planning images (DIR sum plan). We compared cumulative dose parameters between the baseline plan, DVH sum plan and DIR sum plan using equivalent doses in 2 Gy fractions (EQD2).

**Results:**

Individual patients presented relevant increases of near-maximum doses inside the proximal bronchial tree, spinal cord, heart and gastrointestinal OAR when comparing adaptive treatment to the baseline plans. The spinal cord near-maximum doses were significantly increased in the liver patients (D2% median: baseline 6.1 Gy, DIR sum 8.1 Gy, DVH sum 8.4 Gy, p = 0.04; D0.1 cm³ median: baseline 6.1 Gy, DIR sum 8.1 Gy, DVH sum 8.5 Gy, p = 0.04). Three OAR overdoses occurred during adaptive treatment (DIR sum: 1, DVH sum: 2), and four more intense OAR overdoses would have occurred during non-adaptive treatment (DIR sum: 4, DVH sum: 3). Adaptive treatment maintained similar PTV coverages to the baseline plans, while non-adaptive treatment yielded significantly worse PTV coverages in the lung (D95% median: baseline 86.4 Gy, DIR sum 82.4 Gy, DVH sum 82.2 Gy, p = 0.006) and liver patients (D95% median: baseline 87.4 Gy, DIR sum 82.1 Gy, DVH sum 81.1 Gy, p = 0.04).

**Conclusion:**

OAR doses can increase during SMART, so that re-irradiation should be planned based on dose accumulations of the adapted plans instead of the baseline plan. Cumulative dose volume histograms represent a simple and conservative dose accumulation strategy.

**Supplementary Information:**

The online version contains supplementary material available at 10.1186/s13014-023-02284-7.

## Introduction

Re-irradiation has become an important topic in radiation oncology [1]. Innovative systemic treatments enable long-term tumor control in more and more patients with metastatic disease. Especially patients with a low metastatic burden or “oligometastases” have moved into the spotlight of current (radiation) oncology research [2]. They have good chances for long-term tumor control if modern systemic treatments are combined with ablation of all visible metastases [3–6]. Stereotactic ablative body radiotherapy (SABR) can precisely ablate tumors with high single doses in few fractions while sparing neighboring organs-at-risk (OAR). Consequently, SABR enables the non-invasive ablation of oligometastases and its benefits are supported by prospective clinical data in several tumor entities [3–6]. As soon as we pushed the boundaries of long-term tumor control, we also encountered more and more patients where only few metastases show a progression. These cases of “oligoprogression” may again benefit from local ablation of the progressing lesions to postpone initiation or switch of systemic treatments [3, 5, 7–9]. Many oligoprogressions occur in a region that has already been irradiated [10–13], which leads to frequent re-irradiations. Re-irradiation means a new course of radiotherapy to a previously irradiated volume or where the cumulative OAR doses raise concerns about major toxicity [1]. Therefore, the treating radiation oncologist requires detailed knowledge about the doses that have been applied previously. Accordingly, the recent ESTRO consensus on re-irradiation strongly recommends to assess cumulative doses if a high-dose re-irradiation is planned [1].

Magnetic resonance linear accelerators (MR-linacs) allow for MR-imaging before each radiation fraction, with the patient lying on the treatment couch [10, 14]. Thus, the treating team can project the initial treatment plan (baseline plan) on the daily anatomy predict the doses inside the tumor and OAR (predicted plan) and perform online plan adaptation (adapted plan) to account for anatomical changes [10, 14]. When combined with SABR, this approach is termed stereotactic magnetic resonance (MR)-guided online adaptive radiotherapy (SMART) [15]. SMART has taught us that we would sometimes apply doses to the tumor and OAR which are quite different from what we planned due to anatomical changes, i.e. the baseline plans often deviate from the predicted plans. Of course, SMART also yields the opportunity to adapt our plans to such anatomical changes [10, 14–17]. Currently available commercial systems offer online plan adaptation on a daily basis but do not support dose accumulation of all adapted fractions. Therefore, dose accumulation is not performed routinely and its clinical value has not been fully established yet. Most previous works have evaluated the benefits of adaptive treatment based on comparison of single fraction doses [14, 16–20]. Recently, several reports have also evaluated adaptive treatment based on cumulative dose volume histograms (DVH) [19] or cumulative doses created via deformable image registration (DIR) algorithms [21–24]. However, these works have focused on cumulative doses inside the PTV and selected OAR close to the PTV to assess the properties of and/or predict adverse events after SMART (bladder: [21, 24], rectum: [23], stomach & intestines: [22], stomach, intestines & liver: [19]).

In this work, we evaluate doses to the tumor and all relevant OAR during SMART of liver and lung tumors to inform potential re-irradiation. We aim to compare the properties of the baseline plans, cumulative DVHs, and DIR-based dose accumulations.

## Patients and methods

### Patients

We analyzed 18 patients with lung tumors (N = 10, both early-stage non-small cell lung cancer and pulmonary metastases) or liver metastases (N = 8) who received SMART between 02/2020 and 08/2020 inside prospective databases. We have already included these patients in previous comparisons between adapted and predicted plans based on single-doses [14, 20]. Patient and treatment characteristics are summarized in Table 1.

**Table 1:** Patient and treatment characteristics

	Lung (N = 10)	Liver (N = 8)
**Age [years]**	Median: 76 Range: 65–84	Median: 69 Range: 49–77
**Sex**	Male: 8 Female: 2	Male: 3 Female: 5
Location*	Peripheral: 6 Central: 2 Ultracentral: 2	---
**Fractionations**	5 × 10 Gy: 4 8 × 7.5 Gy: 4 10 × 5 Gy: 1 10 × 6 Gy: 1	3 × 15 Gy: 2 5 × 10 Gy: 3 8 × 7.5 Gy: 1 10 × 5 Gy: 2

*liver lesions were considered high-risk due to close proximity to a sensitive OAR or due to large volume

### Treatment planning

Detailed treatment procedures can be found in our previous reports [14, 20]. Briefly, patients were treated at the MRIdian Linac® system (ViewRay Inc., Denver, USA) via step-and-shoot intensity modulated radiotherapy (IMRT). All patients were immobilized with their arms above the head (WingSTEP MR®, Innovative Technologie Völp e.U., Innsbruck, Austria) and underwent pre-treatment simulation at the MR-linac, which included 3D MRI and 2D cineMRI for planning purposes. Moreover, they underwent a planning CT scan (Siemens SOMATOM Confidence®, Siemens Healthcare GmbH, Erlangen, Germany) immediately afterwards.

We defined the gross tumor volume (GTV) as macroscopically visible tumor spread. The GTV was expanded by 2 mm in case of lung tumors and 5 mm in case of liver tumors to obtain the clinical target volume (CTV), thereby respecting anatomical borders and adjacent OAR. The CTV was expanded by 3 mm to obtain the planning target volume (PTV). We chose the dose fractionation according to the risk of side effects. Lung tumors were classified as peripheral (5 × 10 Gy or 8 × 7.5 Gy when PTV touched the thoracic wall), central according to [25] (8 × 7.5 Gy) or ultracentral when the PTV overlapped the proximal bronchial tree (PBT) or esophagus (10 × 5 Gy). Liver tumors were treated with similar dose fractionations based on the intrahepatic location of the metastasis (3 × 15 Gy or 5 × 10 Gy for intrahepatic lesions, 8 × 7.5 Gy for lesions close to the heart and 10 × 5 Gy for lesions adjacent to the small bowel). Usually, we aimed for a 95% coverage of the PTV with the prescribed dose and maximum PTV doses of 125% (154% in case of 3 × 15 Gy). One large liver metastasis (PTV = 373 cm³) required homogeneous dose prescription, so that we aimed for 95% coverage of the PTV by 95% of the prescribed doses and a maximum PTV dose of 107%. Dose constraints for different OARs and fractionations were chosen according to international standards [26, 27] and are given in supplementary Table 1. We prioritized OAR constraints over PTV coverage.

### Radiation treatment

At the beginning of each treatment, patients were immobilized on the treatment couch and underwent 3D MRI. This daily MRI was rigidly registered to the planning MRI based on the GTV contours with a consecutive couch shift for patient positioning. Planning contours and CT-imaging were deformably registered to the daily MRI. The treating team adapted the GTV contours as well as the OAR contours in a region expanding 1 cm craniocaudally and 3 cm in all other directions from the PTV (PTV_expand_) [28]. The PTV_expand_ allows for a fast and robust online adaptation workflow and successfully protects OAR from high doses to small volumes [28], but evaluation of mean doses in organs with large volumes outside the 3 cm sphere may be limited. Subsequently, the baseline plan was re-calculated on the daily anatomy to obtain the predicted plan. The treating physician could initiate plan adaptation using the baseline planning objectives and beam parameters to obtain the adapted plan. Such plan adaptation was mandatory if planning objectives were violated. Lastly, on-table quality assurance was performed [29]. SABR employed gating of the beam during repeated breath holds. For this purpose, the treating team defined a region of interest (ROI) on the 2D cineMRI (e.g. the tumor) and added a 3 mm margin as gating boundary. During irradiation, 2D cineMRI was constantly active and automatically tracked this ROI. Only a small percentage of the ROI was allowed outside the gating boundary (in general 3% to account for technical uncertainties), otherwise the radiation beam was automatically turned off. Subsequently, patients could be instructed to hold their breath to bring the target volume into the intended position.

### Dose accumulation

We imported all baseline plans, predicted plans, and adapted plans together with the respective imaging data (baseline MRI and daily MRIs) and contours (PTV, OAR) into RayStation® 10 A (RaySearch Laboratories AB, Stockholm, Sweden). Since the OAR had only been contoured inside the PTV_expand_ volume, we re-contoured all OAR completely within the whole field-of-view and re-calculated their daily predicted and adapted doses. Firstly, we matched all daily MRIs with their respective baseline MRI using a rigid image registration that based on grey levels. Secondly, we refined this match and employed a deformable image registration (DIR) based on grey levels and contours with all available contours (Raystation’s built-in ANACONDA algorithm [23, 30–32]). Contour-based algorithms have repeatedly outperformed merely grey level-based algorithms [33]. DIR of gastrointestinal (GI) OAR was particularly challenging due to vivid bowel motion and different filling states. Hence, we matched GI OAR with the same deformable image registration (DIR) based on grey levels and contours, but this time only using only the contour of the specific organ. Nevertheless, the intestines still presented major DIR inaccuracies at the caudal border of the field-of-view, so that we created an additional subvolume lying within 8 cm from the PTV in all directions (PTV_expand_ + 5 cm) to represent the most relevant parts for assessment of high doses. Finally, dose distributions of the predicted and adapted plans could be transformed to the planning images via the respective DIR vector fields, and dose accumulation was performed using RayStation’s in-build functionality [32] to obtain a DIR-based dose accumulation. For brevity, we are going to call these DIR sum plans. Furthermore, we summed up the dose volume histograms (DVH) of all predicted or adapted plans on the daily images (i.e. without any registration) to obtain cumulative DVHs for each patient using python version 3.9.12. For brevity, we are going to call these DVH sum plans.

### Statistical analysis

We extracted different dose parameters from baseline plans, predicted plans, adapted plans and respective DIR and DVH sum plans: near-maximum doses to 2% of the volume (D_2%_) and to 0.1 cm³ or 0.5 cm³ of the volume (D_0.1/0.5 cm³_) were extracted for spinal cord, esophagus, heart, PBT and GI OAR. The median dose (D_50%_) was extracted for the lungs, liver and PTV together with the dose to 1500 cm³ of the lung volume (D_1500cm³_) and PTV coverage (D_95%_) (supplementary Table 2). All doses were converted to the equivalent dose in 2 Gy fractions (EQD2) as recommended for assessment of a re-irradiation [1] according to the linear quadratic formula:

$$EQD2=D\bullet \left[\frac{d+ \raisebox{1ex}{$\alpha $}\!\left/ \!\raisebox{-1ex}{$\beta $}\right. }{2+ \raisebox{1ex}{$\alpha $}\!\left/ \!\raisebox{-1ex}{$\beta $}\right.}\right]$$

with the total dose D and single dose d and assuming α/β = 3 for OAR and α/β = 10 for the PTV. These α/β values were chosen according to our clinical routine.

Additionally, we calculated the Dice similarity score and maximum Hausdorff distances (HD) for the DIR of each OAR and each fraction to assess DIR quality. We described the dose parameters, Dice scores and HD using group medians, ranges, scatterplots, and boxplots. We screened each individual patient for increases of OAR doses from the baseline plan, and considered increases > 2 Gy EQD2 (i.e. > 1 normofractionated single dose) clinically relevant. We calculated Friedman’s tests to assess differences of OAR doses between the baseline plan, DIR sum plan and DVH sum plan with and without plan adaptation over all patients in the liver as well as in the lung cohort. To account for multiple testing, p-values were corrected according to the Bonferroni-Holm procedure to maintain a global level of significance α = 0.05. Post-hoc analyses via pairwise two-sided Wilcoxon signed-rank tests were only performed in case of a statistically significant difference. Statistical analyses were performed in python version 3.9.12.

## Results

### Adaptive treatment

Plan adaptation was performed successfully in 118/121 fractions (97.5%). One patient asked to stop adaptation procedures after 7/10 fractions due to back pain.

**Fig. 1:**
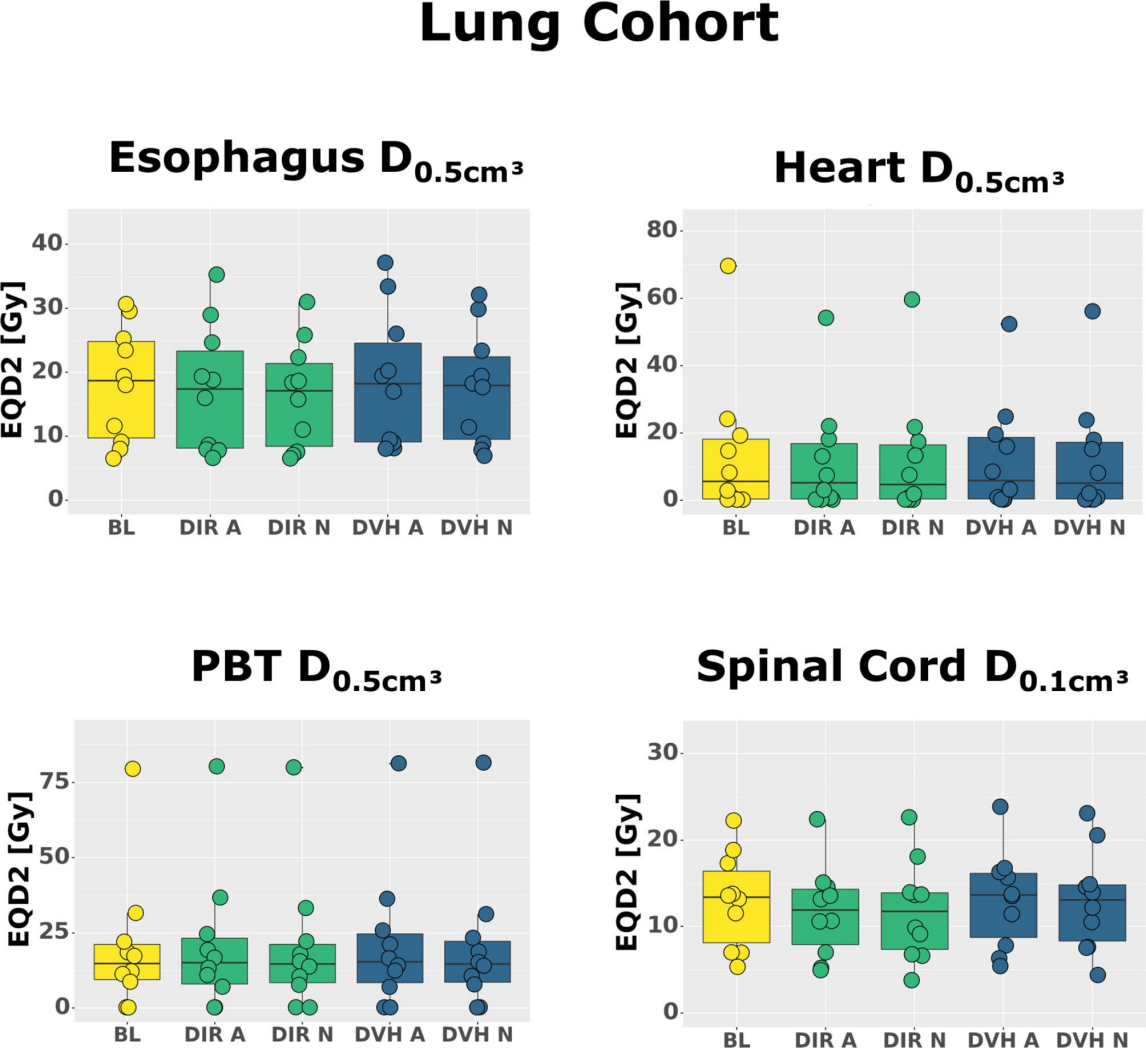
Dose Comparisons in the lung cohort. Comparison of doses in different organs between baseline plans (BL, yellow), deformable image registration-based dose accumulation plans (DIR, green) and cumulative dose volume histogram plans (DVH, blue) for adaptive treatment (A) and non-adaptive treatment (N). All doses were converted to the equivalent dose in 2 Gy (Gy) single doses (EQD2).

**Fig. 2:**
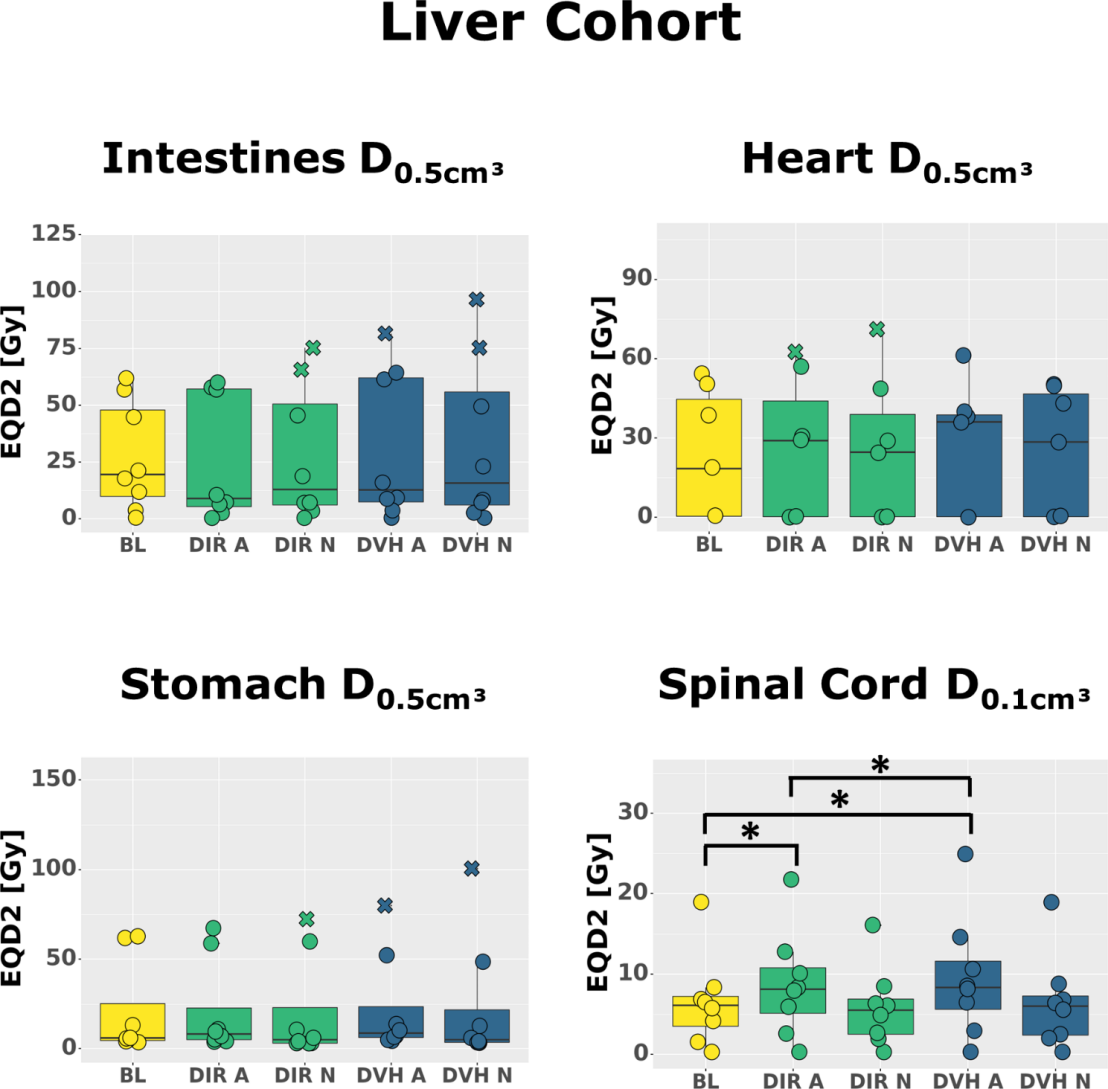
Dose Comparisons in the liver cohort. Comparison of doses in different organs between baseline plans (BL, yellow), deformable image registration-based dose accumulation plans (DIR, green) and cumulative dose volume histogram plans (DVH, blue) for adaptive treatment (A) and non-adaptive treatment (N). All doses were converted to the equivalent dose in 2 Gy (Gy) single doses (EQD2). *: statistically significant with p < 0.05

Firstly, we compared the OAR doses over the whole patient cohorts (Table 2). In this overall comparison, the OAR doses differed only little between the baseline plan, DVH sum plans and DIR sum plans (Figs. 1 and 2), with one exception: Both DVH sum and DIR sum plans yielded significantly increased spinal cord D_2%_ and D_0.1 cm³_ when compared to the baseline plans in the whole liver cohort (D_2%_ median [range]: baseline 6.1 [0.3–18.3] Gy, DIR sum 8.1 [0.3–21.3] Gy, DVH sum 8.3 [0.3–24.0] Gy, p = 0.04; D_0.1 cm³_ median [range]: baseline 6.1 [0.3–18.9] Gy, DIR sum 8.1 [0.3–21.8] Gy, DVH sum 8.3 [0.3–24.9] Gy, p = 0.04), but not in the lung cohort.

**Table 2:** Adaptive treatment: comparison between the baseline plan (BL), deformable image registration-based dose accumulation plan (DIR) and cumulative dose volume histogram plan (DVH).

Lung cohort							
Structure	Metric	Median [Gy EQD 2]			p -value (corr.)	Individual ∆max from baseline [Gy EQD 2]	
		BL	DVH	DIR		DVH (respective DIR)	DIR (respective DVH)
**spinal cord**	**D**_**2%**_	12.6	13.4	11.7	1	**3.1** (1.9)	1.9 (**3.1**)
	**D**_**0.1 cm³**_	13.4	13.6	11.9	1	**2.5** (1.4)	1.4 (**2.5)**
**lungs**	**D**_**50%**_	0.5	0.6	0.5	0.38	0.2 (0.9)	0.9 (0.2)
	**D**_**1500cm³**_	1.7	1.6	2.0	1	0.8 (-0.6) #	1.2 (**-3.1**) #
**heart**	**D**_**2%**_	4.2	3.9	3.4	1	0.7 (0.0) #	0.0 (0.0) #
	**D**_**0.5 cm³**_	5.6	6.0	5.3	0.39	1.3 (-1.6) #	0.2 (0.4) #
**esophagus**	**D**_**2%**_	18.6	17.9	16.5	1	**6.4 (4.8)**	**4.8 (6.4)**
	**D**_**0.5 cm³**_	18.7	18.2	17.4	1	**8.2 (3.7)** #	**4.6 (6.5)** #
**PBT**	**D**_**2%**_	13.6	13.8	13.3	1	**3.5** (1.9) #	**4.9** (1.5) #
	**D**_**0.5 cm³**_	14.8	15.4	15.1	1	**4.7 (5.1)**	**5.1 (4.7)**
**PTV**	**D**_**95%**_	86.4	86.2	85.5	1	0.6 (0.1) #	0.9 (0.5) #
**Liver cohort**							
**spinal cord**	**D**_**2%**_	6.1	8.3	8.1	**0.04**	**8.6 (7.1)**	**7.1 (8.6)**
	**D**_**0.1 cm³**_	6.1	8.3	8.1	**0.04**	**8.8 (7.0)**	**7.0 (8.8)**
**esophagus**	**D**_**2%**_	7.4	8.2	6.9	1	**8.3 (7.3)**	**7.3 (8.3)**
	**D**_**0.5 cm³**_	7.4	8.3	6.9	1	**4.8 (7.4)**	**7.4 (4.8)**
**stomach**	**D**_**2%**_	5.2	5.7	6.4	1	**5.5** (1.1) #	1.4 (0.8) #
	**D**_**0.5 cm³**_	5.8	8.6	8.3	1	**18.1 (-3.0)** #	**4.6 (-10.6)** #
**intestines**	**D**_**2%**_	6.0	5.8	5.14	1	**4.1 (-7.8)** #	0.2 (-0.9) #
	**D**_**0.5 cm³**_	19.5	12.6	8.89	1	**19.7** (-1.8) #	**12.1 (19.5)** #
**heart**	**D**_**2%**_	3.9	10.9	8.4	1	**7.0 (4.5)** #	**4.7 (2.8)** #
	**D**_**0.5 cm³**_	18.5	36.1	28.9	1	**22.6 (18.2)**	**18.2 (22.6)**
**liver**	**D**_**50%**_	0.9	1.0	1.35	1	**4.2 (2.1)**	**2.1 (4.2)**
**PTV**	**D**_**95%**_	87.4	85.6	86.27	1	**9.5 (9.1)**	**9.1 (9.5)**

EQD2: equivalent dose in 2 Gy fractions, p-value (corr.): Bonferroni-Holm corrected p-value for overall comparison of the different plans (values < 0.05 in bold), ∆max: maximum difference to the baseline plan (all absolute differences > 2 Gy EQD2 in bold, #: DVH and DIR find maximum difference in different patients), D_XX%_: dose to XX% of the volume, D_cm³_: dose to XX cm³ of the volume

Secondly, we compared the OAR doses in each patient individually and identified relevant (> 2 Gy EQD2) increases of near-maximum doses inside the PBT, spinal cord, heart and GI OAR (supplementary Fig. 1, supplementary Fig. 2). Usually, both DIR and DVH sum plans demonstrated such increases, with DVH sum plans indicating larger increases. Either DVH or DIR sum plans, but not both, demonstrated violations of dose constraints in three cases.

In the following, one can find the largest individual dose increases in Gy EQD2 according to the DVH sum plan (respective DIR sum plan in brackets) (Table 2 and supplementary Table 3):

- the spinal cord and D_0.1 cm³_ exceeded the baseline plan by up to 2.5 (1.4) Gy in the lung cohort and by up to 8.8 (7.0) Gy in the liver cohort.
- The esophagus D_0.5 cm³_ exceeded the baseline plan by up to 8.2 (3.7) Gy in the lung cohort and by up to 4.8 (7.4) Gy in the liver cohort.
- In the liver cohort, the heart D_0.5 cm³_ exceeded the baseline plan by up to 22.6 (18.2) Gy, the stomach D_0.5 cm³_ exceeded the baseline plan by up to 18.1 (-3.0) Gy, and the intestinal D_0.5 cm³_ exceeded the baseline plan by up to 19.7 (-1.8) Gy. We found dose constraint violations in the heart (#16: DVH sum: − 11.2 Gy, DIR sum: + 11.1 Gy) and in the stomach (#16: DVH sum: + 9.9 Gy, DIR sum: − 11.2 Gy) of one patient, and in the intestines (#18: DVH sum: + 11.5 Gy, DIR sum: − 10.0 Gy) of another patient. Analysis of intestinal subvolumes confirmed these results (supplementary Table 3).
- In the lung cohort, the PBT D_0.5 cm³_ exceeded the baseline plan by up to 4.7 (5.1) Gy.

Plan adaptation managed to maintain a good PTV coverage both in the lung cohort (D_95% EQD2_ median [range]: baseline 86.4 [63.4–89.3] Gy, DIR sum 85.5 [63.8–90.0] Gy, DVH sum 86.2 [63.6–89.7] Gy, p = 1.0) and in the liver cohort (D_95%_ median [range]: baseline 87.4 [58.5–136.9] Gy, DIR sum 86.3 [57.4–133.9] Gy, DVH sum 85.6 [57.3–100.1] Gy, p = 1.0) without a statistically significant difference to the baseline plans. In the liver cohort, we even observed one case with an increase of the PTV D_95%_ by 9.5 (9.1) Gy (supplementary Fig. 3).

### Non-adaptive treatment

We used the predicted plans to simulate a theoretical non-adaptive treatment.

Again, we started with a comparison of OAR doses over the whole patient cohorts (Table 3). Similar to adaptive treatment, the OAR doses differed only little between the baseline plan, DVH sum plans and DIR sum plans (Figs. 1 and 2). In contrast to adaptive treatment, we did not find statistically significant differences between the near-maximum doses inside the spinal cord or any other OAR.

**Table 3:** Non-adaptive treatment: comparison between the baseline plan (BL), deformable image registration-based dose accumulation plan (DIR) and cumulative dose volume histogram plan (DVH).

Lung cohort							
Structure	Metric	Median [Gy EQD 2]			p -value (corr.)	Individual ∆max from baseline [Gy EQD 2]	
		BL	DVH	DIR		DVH (respective DIR)	DIR (respective DVH)
**spinal cord**	**D**_**2%**_	12.6	12.8	11.2	0.64	1.8 (0.9)	0.9 (1.8)
	**D**_**0.1 cm³**_	13.4	13.1	11.8	0.64	1.7 (-0.7)	0.5 (1.4) #
**lungs**	**D**_**50%**_	0.5	0.5	0.5	0.38	0.1 (0.0)	0.7 (0.1) #
	**D**_**1500cm³**_	1.7	1.7	1.9	1	0.5 (-0.7)	0.6 (-0.4) #
**heart**	**D**_**2%**_	4.2	3.6	3.2	1	0.0 (0.0)	0.0 (0.0)
	**D**_**0.5 cm³**_	5.6	5.2	4.8	1	0.5 (-1.4)	0.0 (0.1) #
**esophagus**	**D**_**2%**_	18.7	17.6	16.4	1	**2.9** (0.7)	0.7 (**2.9**)
	**D**_**0.5 cm³**_	18.7	18.0	17.1	1	**4.6** (0.6)	0.6 (**4.6**)
**PBT**	**D**_**2%**_	13.6	13.2	13.1	1	1.9 (-0.1)	1.0 (1.1) #
	**D**_**0.5 cm³**_	14.8	14.7	14.7	1	**2.1** (0.5)	1.6 (-0.4) #
**PTV**	**D**_**95%**_	86.4	82.2	82.4	**0.006**	-1.5 (-1.3)	-1.2 (-2.0) #
**Liver cohort**							
**spinal cord**	**D**_**2%**_	6.1	5.9	5.3	1	1.0 (1.1)	1.1 (1.0)
	**D**_**0.1 cm³**_	6.1	6.0	5.5	1	1.0 (1.1)	1.1 (1.0)
**esophagus**	**D**_**2%**_	7.4	9.6	8.9	1	**2.2** (1.4)	1.4 (**2.2**)
	**D**_**0.5 cm³**_	7.4	9.7	8.8	1	**2.3** (1.4)	1.4 (**2.3**)
**stomach**	**D**_**2%**_	5.2	4.2	4.5	1	**16.1 (10.0)**	**10.0 (16.1)**
	**D**_**0.5 cm³**_	5.8	5.0	5.1	1	**38.8 (10.5)**	**10.5 (38.8)**
**intestines**	**D**_**2%**_	6.0	6.8	5.3	0.93	**11.3** (-0.8)	0.0 (0.0) #
	**D**_**0.5 cm³**_	19.5	15.7	13.0	1	**34.6 (13.3)**	**13.3 (34.6)**
**heart**	**D**_**2%**_	3.9	7.8	6.0	1	**3.8 (**2.0**)**	**4.4 (2.6)** #
	**D**_**0.5 cm³**_	18.5	28.5	24.6	1	**11.7 (9.9)**	**20.6** (-0.7) #
**liver**	**D**_**50%**_	0.9	0.9	1.1	1	**2.4** (0.8)	0.8 (**2.4**)
**PTV**	**D**_**95%**_	87.4	75.1	82.1	**0.04**	**-3.9 (-2.9)**	0.7 (**-4.1**) #

EQD2: equivalent dose in 2 Gy fractions, p-value (corr.): Bonferroni-Holm corrected p-value for overall comparison of the different plans (values < 0.05 in bold), ∆max: maximum difference to the baseline plan (all absolute differences > 2 Gy EQD2 in bold, #: DVH and DIR find maximum difference in different patients), D_XX%_: dose to XX% of the volume, D_cm³_: dose to XX cm³ of the volume

**Fig. 3:**
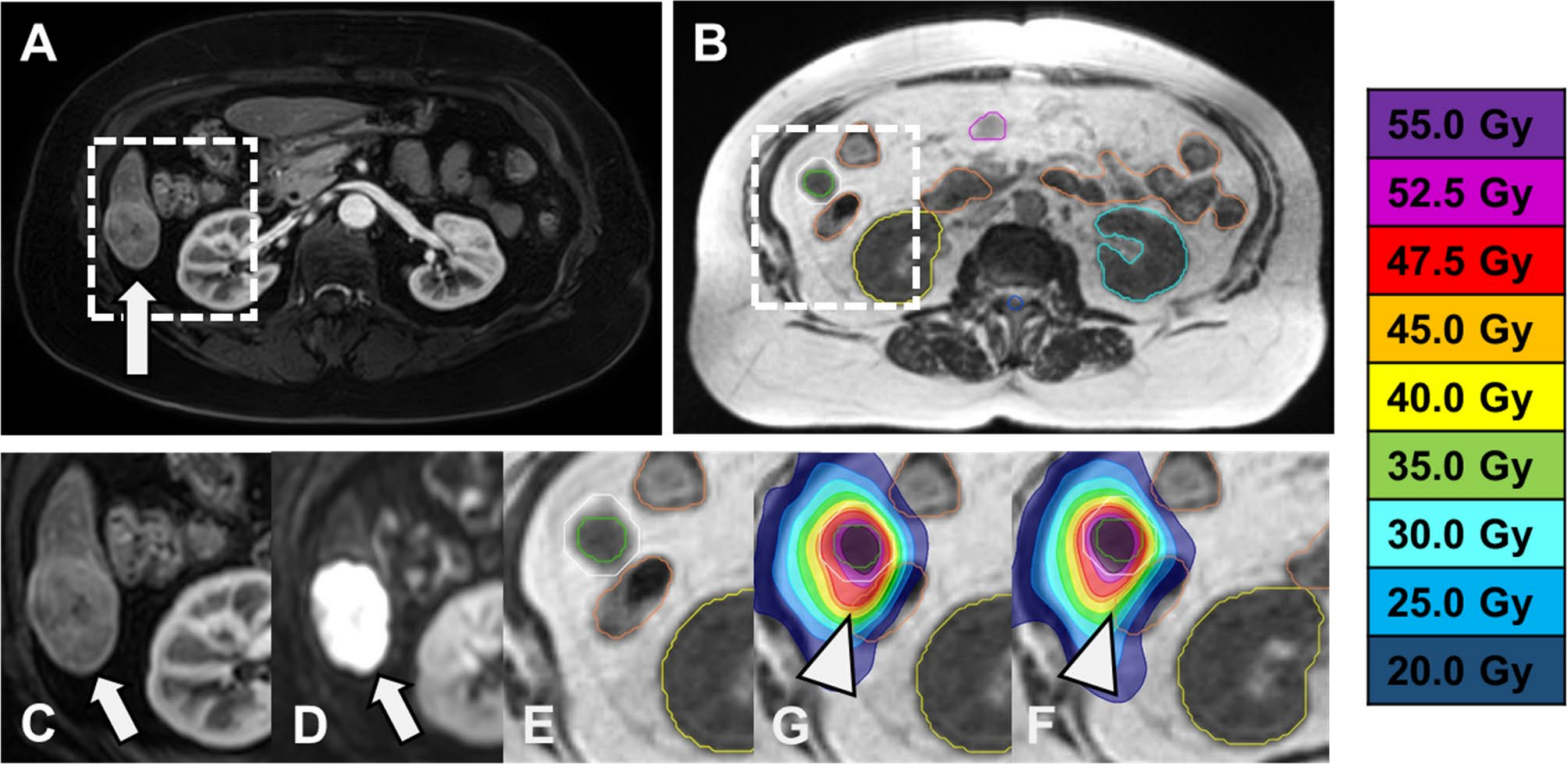
Case study: SMART of a liver metastasis. **(A)** Diagnostic T1w contrast enhanced (CE) MRI with liver metastasis in the caudal segment VI (white arrow). **(B)** Baseline imaging at the MR-Linac **(**TrueFISP). **(C) – G)** Zoom into the white frames in A) and B), with **C)** Diagnostic T1w CE MRI, **(D)** Diagnostic diffusion-weighted MRI (b = 600 s/mm²), **(E)** MR-Linac TrueFISP, **(F)** deformable image registration (DIR)-based dose accumulation of predicted plans (theoretical non-adaptive treatment) with overdose inside the intestines (white arrowhead) and **(G)** DIR-based dose accumulation of adapted plans with protection of the intestines (white arrowhead)

Then, we compared the OAR doses in each patient individually. Similar to adaptive treatment, we found relevant individual increases of the near-maximum dose inside the PBT, heart and GI OAR (supplementary Fig. 1, supplementary Fig. 2). Again, both DIR and DVH sum plans demonstrated such increases, with DVH sum plans indicating larger increases. We found four cases of dose constraint violations. In contrast to adaptive treatment, the overdoses were mostly confirmed by both DVH and DIR sum plans, and overdoses were higher than in the respective adaptive treatments. Figure 3 shows the case study of a patient with considerable overdoses in the intestines in non-adaptive treatment.

In the following, one can find the largest individual dose increases in Gy EQD2 according to the DVH sum plan (respective DIR sum plan in brackets) (Table 3 and supplementary Table 4):

- The esophagus D_0.5 cm³_ (EQD2) exceeded the baseline plan by up to 4.6 (0.6) Gy in the lung cohort and by up to 2 2.3 (1.4) Gy in the liver cohort.
- In the liver cohort, the heart D_0.5 cm³_ (EQD2) exceeded the baseline plan by up to 11.7 (9.9) Gy, the stomach D_0.5 cm³_ exceeded the baseline plan by up to 38.8 (10.5) Gy, and the intestinal D_0.5 cm³_ exceeded the baseline plan by up to 34.7 (13.4) Gy. We found dose constraint violations in the heart (#16: DVH sum: -1.2 Gy, DIR sum: + 20.1 Gy,) and in the stomach (#16: DVH sum: + 30.6 Gy, DIR sum: + 2.3 Gy) of one patient as well as in the intestines of two more patients (#13: DVH sum: + 11.3 Gy, DIR sum: + 1.7 and #18: DVH sum: + 26.5 Gy, DIR sum: +5.2 Gy).
- In the lung cohort, the PBT D_0.5 cm³_ (EQD2) exceeded the baseline plan by up to 2.1 (0.5) Gy.

PTV coverage was compromised inside the predicted plans, which reached statistical significance both in the lung cohort (D_95%_ median [range]: baseline 86.4 [63.4–89.3] Gy, DIR sum 82.4 [61.3–88.0] Gy, DVH sum 82.4 [61.3–88.0] Gy, p = 0.006) and in the liver cohort (D_95%_ median [range]: baseline 87.4 [58.5–136.9] Gy, DIR sum 82.1 [52.4–110.9] Gy, DVH sum 75.1 [50.7–91.0] Gy, p = 0.04) (supplementary Fig. 3).

### Deformable image Registration

We evaluated the DIR quality with Dice scores and maximum Hausdorff distances (HD). The lung cohort reached excellent Dice scores ≥ 0.9 in all OAR except for the esophagus (esophagus: 0.89 ± 0.07, heart: 0.99 ± 0.01, lungs: 0.97 ± 0.01, PBT: 0.94 ± 0.04, spinal cord: 0.92 ± 0.03) and corresponding HD < 2 mm except for the lungs with their larger organ volume (esophagus: 1.3 ± 1.0 mm, heart: 0.6 ± 0.3 mm, lungs: 2.9 ± 0.8 mm, PBT: 1.1 ± 0.8 mm, spinal cord: 1.3 ± 1.2 mm) (supplementary Tables 5, supplementary Fig. 4).

The liver cohort reached excellent Dice scores ≥ 0.9 in the heart, kidneys, liver and spinal cord (heart: 0.98 ± 0.01 mm, liver: 0.96 ± 0.01, kidneys: each 0.97 ± 0.01, spinal cord: 0.90 ± 0.04), while fair Dice scores > 0.7 were reached in GI OAR (esophagus: 0.74 ± 0.12, intestines: 0.74 ± 0.18, stomach: 0.88 ± 0.12). This was reflected by the HD (heart: 0.9 ± 0.6, liver: 2.6 ± 1.7 mm, kidneys: right 0.8 ± 0.4 mm and left 0.6 ± 0.3 mm, spinal cord: 1.5 ± 1.3 mm, esophagus: 6.0 ± 3.8 mm, intestines: 4.3 ± 1.5 mm, stomach: 2.1 ± 1.4 mm). Dice scores and HD did not improve when analyzing intestinal subvolumes (Dice: 0.7 ± 0.34, HD: 3.4 ± 1.8 mm) (supplementary Tables 5, supplementary Fig. 5).

## Discussion

SMART yields an excellent opportunity to apply ablative doses to tumors in vulnerable locations because adaptive treatment maintains high PTV coverage while protecting radiosensitive OAR [10, 16, 18]. Here, we investigate cumulative doses inside all OAR compared to the baseline plan to inform potential re-irradiation after SMART.

Our results suggest relevant dose increases in individual patients during adaptive treatment, even though we could not find statistically significant deviations over the whole cohorts for most OAR. Expectedly, such dose increases may occur in OAR close to the PTV and are probably caused by organ movements towards the PTV. For example, we found an increased esophagus D_0.5 cm³_ of up to 8.4 Gy for an ultracentral lung tumor and of up to 5.0 Gy for a liver metastasis in the left lobe close to the cardia. Similarly, relevant increases of near-maximum doses occurred in the stomach or intestines of liver patients, where we even found dose constraint violations in three cases. In one patient, the treating team had accepted increased heart D_0.5 cm³_ in few adapted fractions. Overall, the DVH sum plan complied with the dose constraint, but the DIR sum plan suggested a dose constraint violation. Other dose constraint violations were found in the DVH sum of the stomach and intestines D_0.5 cm³_ in two different patients, although all clinical plans had complied with the respective dose constraints. We recontoured all OAR, which might have had a large impact on the small organ volumes involved in the D_0.5 cm³_, especially close to the PTV. Moreover, DIR sum plans did not confirm these dose constraint violations. Conversely, dose increases were higher in the predicted plans and were confirmed by both the DVH and DIR sum plans. Hence, plan adaptation reduced overdoses and protected the OAR, which agrees with previous single dose [10, 14, 16, 18] and cumulative dose analyses [19]. In contrast to previous studies [14, 16], we observed only one single fraction dose constraint violation in the lung cohort, which did not lead to a violation of sum plans. This can be explained with the large number of peripheral lung tumors that show less pronounced dosimetry benefits during adaptive treatment [14, 34].

We also found relevant dose increases in OAR with some distance to the target volumes, particularly the spinal cord (up to + 9 Gy EQD2). Spinal cord near-maximum doses were even statistically significantly increased due to adaptive treatment, but not non-adaptive treatment, over all liver patients. Probably the treating team systematically decided to accept an increase of spinal cord doses, all below the respective dose constraints, to cope with the conflict of reduced PTV coverage versus nearby OAR overdoses. Of course, such an approach seems reasonable during the current treatment. Yet, information about potential dose increases in such a critical OAR must be available for potential re-treatments. Furthermore, our institutional practice is to avoid strong increases of OAR doses during plan adaptation whenever possible. Approaches where critical OAR dose constraints represent the only limit might produce more pronounced increases of OAR doses far from the PTV.

Together with recent developments in innovative systemic treatments, re-irradiation is gaining importance in clinical practice [1]. Modern medical treatments can increasingly prevent ubiquitous tumor progression, so that local ablative treatment of few progressive lesions (oligoprogression) has become an important need in oncology. Radiotherapy, especially adaptive stereotactic treatment, yields effective local tumor ablation and is generally well tolerated. However, oligoprogressions frequently (re-)occur at anatomical sites which have already been irradiated [10–13]. Here, the challenge is to avoid cumulative overdoses to OAR. Hence, the ESTRO consensus on re-irradiation recommends cumulative dose assessments [1]. Our findings show that such cumulative dose assessments are not trivial after SMART has been performed at a certain anatomic site. Baseline plans are readily available, but do not inform about OAR dose increases due to anatomical changes at all. DVH sum plans simply add the values of all DVHs, so that they are easy to calculate from all adapted plans. In this analysis, DVH sum plans usually yielded stronger increases of near-maximum doses than DIR sum plans. Since DVH sum plans assume that image voxels do not move and thus always assign the highest dose area to the same OAR subvolume, they tend towards high near-maximum doses in OARs (“worst case”). However, such non-deformable dose accumulation may not only strongly overestimate, but sometimes also underestimate doses in OAR with highly varying positions and volumes, particularly GI OAR. Here, DIR-based dose accumulation should yield more accurate estimates. In our analysis, DIR sum plans usually confirmed the trend seen in DVH sum plans with a lower amplitude. Considerable disagreements occurred in the stomach and intestines in few cases, sometimes even showing dose increases where the other showed a dose decrease. Notably, DIR quality and thus reliability of DIR sum plans was not optimal for stomach and intestines in our analysis despite specific DIR for GI OAR. The respective Dice scores of 0.7–0.8 did not quite reach the recommended level > 0.8 [35]. Besides, even high Dice scores and small HD do not guarantee correct matching of organ subvolumes. All in all, the true near-maximum doses of our cohorts probably lie somewhere in between the DIR and DVH sum plans. From a clinical perspective, the DVH sum plans seem simple and straightforward with generally conservative results. Conversely, DIR sum plans deal with organ motion and deformation, but require full digital versions of all adapted plans and sophisticated DIR algorithms that still face limitations for GI OAR. Nevertheless, DIR allow for calculation of 3D cumulative dose distributions, which are desirable according to the ESTRO consensus on re-irradiation [1]. Innovative developments may improve DIR reliability. For example, Large Deformation Diffeomorphic Metric Mapping (LDDMM) showed high Dice scores in the upper abdomen in a recent study [22]. However, such algorithms are not commercially available yet and may have their shortcomings as well. In the future, different DIR algorithms may be used for different tasks / organ sites, and the algorithms should include uncertainty measures such as confidence bands to quantify their potential bias [36].

Dose accumulation strategies may become a sophisticated future approach not only to inform re-irradiation, but also to guide adaptive-treatment itself via dose accumulation up to the current fraction of the day [22]. Our results suggest that adaptive treatment leads to dynamical changes of dose distributions in some patients, which often reflect the underlying anatomical changes. Since current clinical routine does not implement dose accumulation strategies, clinicians perform a daily balancing act. In specific, dose accumulation can be used as a tool to better balance PTV coverage and OAR dose constraints. For instance, an OAR may lie close to the PTV only during one or two fractions. Thus, it could be possible to maintain high PTV coverage during the whole treatment, with moderate overdoses to this OAR in one or two single fractions but overall compliance with dose constraints in the cumulative plan. In this context, current OAR dose constraints already contain uncertainties of dose application inherent to hitherto non-adaptive radiation techniques. It might be useful to develop specific dose constraints for adaptive treatment, which rely on precise adaptation of the dose to the daily anatomy. Finally, a broader availability of reliable dose accumulation strategies could even be applied in non-adaptive image-guided RT and allow less conservative OAR dose constraints in general.

PTV coverage could be maintained, and in some cases even improved, with adaptive treatment. This agrees with many previous reports [10, 14, 16, 23]. Hence, non-adaptive treatment led to significantly decreased D_95%_ both in the lung (approx. -4 Gy EQD2) and liver patients (approx. -5 to -6 Gy EQD2).

We would like to acknowledge several limitations of our work. We analyzed a heterogeneous patient cohort with peripheral and central lung tumors as well as liver tumors in different locations. Therefore, statistically significant results were difficult to obtain over the whole cohorts. Moreover, we re-contoured the OAR over the whole field-of-view to optimize dose estimations, which might not be possible in a clinical scenario. On the one hand, DIR worked well with excellent DICE scores and small HD for OAR with moderate interfractional changes, e.g. the spinal cord, heart or liver. On the other hand, DIR were limited in GI OAR, which may have limited the DIR-based dose accumulations in GI OAR. We used different approaches to optimize DIR quality: (1) a grey level- and contour-based algorithm [23, 30, 31], (2) specific DIR based on the GI OAR contours only and (3) subvolume analyses. Nevertheless, the remaining inaccuracies may have led to relevant bias when considering the small OAR volumes used to measure near-maximum doses.

## Conclusions

Organ-at-risk (OAR) doses can increase considerably during MR-guided adaptive treatment: (1) OAR can move towards the target volume or (2) the treating team deliberately increases doses inside low dose areas to strike the balance between adequate PTV coverage and protection of OAR inside the high dose area. Therefore, planning of a re-irradiation should be based on dose accumulations of the adapted plans instead of the baseline plan, whenever possible. Currently, cumulative dose volume histograms represent a simple and generally conservative dose accumulation strategy, while further development of deformable image registration (DIR)-based approaches is desirable in the future.

## Electronic supplementary material

Below is the link to the electronic supplementary material.

Supplementary Material 1

## Data Availability

All underlying patient data and radiation plans cannot be made available due to local ethical and legal requirements.
