# Supplementary material for: Comparison of different dose accumulation strategies to estimate organ doses after stereotactic magnetic resonance-guided adaptive radiotherapy
Source: Radiat Oncol. 2023 May 29;18:92. doi: 10.1186/s13014-023-02284-7 (PMC10228097; doi:10.1186/s13014-023-02284-7)

**Supplementary Table 1.** **Institutional dose constraints.**

|  | **3 Fractions** | **5 Fractions** | **8 Fractions** | **10 Fractions** |
| --- | --- | --- | --- | --- |
| **Spinal Cord** |  |  |  |  |
| 0.1 cm³ | < 21.6 Gy | < 27.0 Gy | < 32.0 Gy | < 35.0 Gy |
| **Esophagus** |  |  |  |  |
| 0.5 cm³ | < 25.2 Gy | < 34.0 Gy | < 40.0 Gy | < 43.5 Gy |
| **PBT** |  |  |  |  |
| 0.5 cm³ | < 32 Gy | < 35.0 Gy | < 44.0 Gy | < 100 % * |
| **Heart** |  |  |  |  |
| 0.5 cm³ | < 26.0 Gy | < 29.0 Gy | < 60.0 Gy | < 66.0 Gy |
| **Lung** |  |  |  |  |
| 1500 cm³ | < 10.5 Gy | < 12.5 Gy | < 14.5 Gy | < 15.5 Gy |
| **Liver** |  |  |  |  |
| Mean | < 12.0 Gy | < 15.2 Gy | < 18.0 Gy | < 19.5 Gy |
| **Kidneys** |  |  |  |  |
| Mean | < 8.5 Gy | < 10.0 Gy | < 11.5 Gy | < 12.0 Gy |
| **Stomach** |  |  |  |  |
| 0.5 cm³ | < 22.2 Gy | < 35.0 Gy | < 40.0 Gy | < 42.5 Gy |
| **Intestines** |  |  |  |  |
| 0.5 cm³ | < 25.2 Gy | < 35.0 Gy | < 40.0 Gy | < 43.5 Gy |

Gy: Gray, PBT: proximal bronchial tree. *Related to the prescribed dose.

**Supplementary Table 2. Analysis parameters.**

| Cohort | Structure | Dosimetry Parameter |
| --- | --- | --- |
| Both | Spinal cord | D_2%_, D _0.1cm³_ |
|  | Esophagus | D_2%_, D _0.5cm³_ |
|  | PTV | D_95%_ |
| Lung | Heart | D_2%_, D _0.5cm³_ |
|  | PBT | D_2%_, D _0.5cm³_ |
|  | Lungs | D_50%_, D _1500cm³_ |
| Liver | Stomach | D_2%_, D _0.5cm³_ |
|  | Intestines | D_2%_, D _0.5cm³_ |
|  | Kidneys | D_50%_ |
|  | Liver | D_50%_ |

D_XX%_: dose to XX% of the volume, D_cm³_: dose to XX cm³ of the volume

**Supplementary Table 3.** **Full dose comparison table for adaptive treatment. All values are given in Gy (EQD2).**

| **Lung Cohort** | | **Baseline** | | **DIR sum** | | | **DVH sum** | | |  |  |  |  |
| --- | --- | --- | --- | --- | --- | --- | --- | --- | --- | --- | --- | --- | --- |
|  |  | **Median** | **Range** | **Median** | **Range** | **∆max** | **Median** | **Range** | **∆max** | **p (corr.)** | **p (DIR-BL)** | **p (DVH-BL)** | **p (DIR-DVH)** |
| **spinal cord** | **D_2%_** | 12.64 | 4.51 - 21.67 | 11.71 | 4.75 - 21.94 | 1.91 | 13.36 | 5.17 - 23.13 | **3.08** | 1 | --- | --- | --- |
|  | **D_0.1cm³_** | 13.38 | 5.35 - 22.26 | 11.92 | 4.97 - 22.41 | 1.38 | 13.64 | 5.44 - 23.85 | **2.49** | 1 | --- | --- | --- |
| **lungs** | **D_50%_** | 0.48 | 0.21 - 2.61 | 0.49 | 0.22 - 2.34 | 0.87 | 0.55 | 0.21 - 2.21 | 0.17 | 0.38 | --- | --- | --- |
|  | **D_1500cm³_** | 1.68 | 0.4 - 8.27 | 2.02 | 0.51 - 9.46 | 1.19 | 1.58 | 0.48 - 8.63 | 0.76 | 1 | --- | --- | --- |
| **heart** | **D_2%_** | 4.15 | 0.2 - 31.73 | 3.44 | 0.21 - 26.18 | 0.04 | 3.87 | 0.21 - 27.0 | 0.72 | 1 | --- | --- | --- |
|  | **D_0.5cm³_** | 5.61 | 0.24 - 69.62 | 5.28 | 0.24 - 54.21 | 0.16 | 5.93 | 0.26 - 52.35 | 1.31 | 0.39 | --- | --- | --- |
| **esophagus** | **D_2%_** | 18.55 | 6.19 - 31.94 | 16.5 | 6.36 - 36.73 | **4.78** | 17.91 | 7.84 - 38.33 | **6.38** | 1 | --- | --- | --- |
|  | **D_0.5cm³_** | 18.7 | 6.53 - 30.67 | 17.41 | 6.63 - 35.26 | **4.59** | 18.23 | 8.06 - 37.13 | **8.15** | 1 | --- | --- | --- |
| **PBT** | **D_2%_** | 13.59 | 0.21 - 76.88 | 13.3 | 0.22 - 77.39 | **4.89** | 13.82 | 0.23 - 79.0 | **3.47** | 1 | --- | --- | --- |
|  | **D_0.5cm³_** | 14.78 | 0.22 - 79.53 | 15.05 | 0.23 - 80.36 | **5.12** | 15.37 | 0.23 - 81.35 | **4.71** | 1 | --- | --- | --- |
| **PTV** | **D_95%_** | 86.35 | 63.42 - 89.31 | 85.47 | 63.78 - 90.04 | 0.86 | 86.19 | 63.56 - 89.69 | 0.62 | 1 | --- | --- | --- |
| **Liver Cohort** | | | | | | | | | | | | | |
| **spinal cord** | **D_2%_** | 6.05 | 0.3 - 18.32 | 8.1 | 0.31 - 21.31 | **7.07** | 8.3 | 0.31 - 24.01 | **8.56** | **0.04** | **0.02** | **0.008** | **0.008** |
|  | **D_0.1cm³_** | 6.13 | 0.3 - 18.94 | 8.13 | 0.31 - 21.76 | **7.02** | 8.33 | 0.32 - 24.92 | **8.84** | **0.04** | **0.02** | **0.008** | **0.008** |
| **esophagus** | **D_2%_** | 7.42 | 0.23 – 54.81 | 6.90 | 0.25 - 61.73 | **7.28** | 8.17 | 0.26 - 62.74 | **8.29** | 1 | --- | --- | --- |
|  | **D_0.5cm³_** | 7.42 | 0.23 – 54.81 | 6.88 | 0.24 - 60.22 | **7.38** | 8.28 | 0.25 - 57.65 | **4.81** | 1 | --- | --- | --- |
| **stomach** | **D_2%_** | 5.2 | 2.99 - 49.29 | 6.41 | 2.79 - 46.45 | 1.44 | 5.65 | 2.79 - 39.33 | **5.46** | 1 | --- | --- | --- |
|  | **D_0.5cm³_** | 5.84 | 3.52 - 62.73 | 8.28 | 3.83 - 67.27 | **4.55** | 8.61 | 4.31 - 79.9 | **18.11** | 1 | --- | --- | --- |
| **intestines** | **D_2%_** | 5.95 | 0.33 - 33.19 | 5.14 | 0.29 - 30.67 | 0.18 | 5.75 | 0.29 - 33.58 | **4.11** | 1 | --- | --- | --- |
|  | **D_0.5cm³_** | 19.49 | 0.47 - 61.87 | 8.89 | 0.36 - 60.04 | **12.09** | 12.61 | 0.41 - 81.54 | **19.67** | 1 | --- | --- | --- |
| **intestines partial** | **D_2%_** | 10.18 | 0.41 - 44.54 | 5.28 | 0.39 - 43.82 | **5.33** | 10.86 | 0.42 - 46.24 | **5.28** | 1 | --- | --- | --- |
|  | **D_0.5cm³_** | 18.76 | 0.46 - 61.87 | 10.2 | 0.43 – 60.08 | **15.30** | 15.95 | 0.5 - 81.54 | **19.67** | 1 | --- | --- | --- |
| **heart** | **D_2%_** | 3.93 | 0.18 - 27.83 | 8.4 | 0.19 - 19.41 | **4.67** | 10.91 | 0.2 - 23.32 | **6.98** | 1 | --- | --- | --- |
|  | **D_0.5cm³_** | 18.45 | 0.19 - 54.71 | 28.9 | 0.21 - 62.18 | **18.22** | 36.1 | 0.22 - 61.46 | **22.57** | 1 | --- | --- | --- |
| **kidney L** | **D_50%_** | 0.29 | 0.16 - 1.46 | 0.29 | 0.18 - 1.0 | 0.37 | 0.3 | 0.18 - 0.98 | 0.2 | 1 | --- | --- | --- |
| **kidney R** | **D_50%_** | 0.45 | 0.19 - 4.9 | 0.56 | 0.2 - 5.23 | 0.33 | 0.51 | 0.19 - 5.25 | 0.35 | 0.13 | --- | --- | --- |
| **liver** | **D_50%_** | 0.9 | 0.37 - 9.99 | 1.35 | 0.44 - 12.07 | **2.08** | 1.04 | 0.43 - 14.18 | **4.18** | 1 | --- | --- | --- |
| **PTV** | **D_95%_** | 87.38 | 58.45 - 136.87 | 86.27 | 57.42 - 133.93 | **9.13** | 85.58 | 57.31 - 100.05 | **9.47** | 1 | --- | --- | --- |

Baseline: initial plan on initial anatomy, DIR sum: dose accumulation via deformable image registration, DVH sum: dose accumulation via dose volume histogram summation, D_XX%_: dose to XX% of the volume, D_cm³_: dose to XX cm³ of the volume, ∆max: maximum individual difference to the baseline plan, p corr.: Bonferroni-Holm corrected p-value, p (DIR-BL): p-value for post-hoc comparison of DIR sum with the baseline plan, p (DVH-BL): p-value for post-hoc comparison of DVH sum with the baseline plan, p (DIR-DVH): p-value for post-hoc comparison of DIR sum with DVH sum.

**Supplementary Table 4. Full dose comparison table for non-adaptive treatment. All values are given in Gy (EQD2).**

| **Lung Cohort** | | **Baseline** | | **DIR sum** | | | **DVH sum** | | |  |  |  |  |
| --- | --- | --- | --- | --- | --- | --- | --- | --- | --- | --- | --- | --- | --- |
|  |  | **Median** | **Range** | **Median** | **Range** | **∆max** | **Median** | **Range** | **∆max** | **p (corr.)** | **p (DIR-BL)** | **p (DVH-BL)** | **p (DIR-DVH)** |
| **spinal cord** | **D_2%_** | 12.64 | 4.51 - 21.67 | 11.18 | 3.62 - 22.08 | 0.9 | 12.77 | 4.13 - 22.28 | 1.8 | 0.64 | --- | --- | --- |
|  | **D_0.1cm³_** | 13.38 | 5.35 - 22.26 | 11.77 | 3.81 - 22.63 | 0.5 | 13.07 | 4.41 - 23.1 | 1.7 | 0.64 | --- | --- | --- |
| **lungs** | **D_50%_** | 0.48 | 0.21 - 2.61 | 0.49 | 0.22 - 2.18 | 0.7 | 0.54 | 0.22 - 2.12 | 0.1 | 0.38 | --- | --- | --- |
|  | **D_1500cm³_** | 1.68 | 0.4 - 8.27 | 1.93 | 0.45 - 8.45 | 0.55 | 1.66 | 0.42 - 8.33 | 0.5 | 1 | --- | --- | --- |
| **heart** | **D_2%_** | 4.15 | 0.2 - 31.73 | 3.24 | 0.21 - 29.61 | 0.02 | 3.59 | 0.21 - 29.29 | 0.0 | 1 | --- | --- | --- |
|  | **D_0.5cm³_** | 5.61 | 0.24 - 69.62 | 4.75 | 0.23 - 59.65 | 0.02 | 5.15 | 0.25 – 56.15 | 0.5 | 1 | --- | --- | --- |
| **esophagus** | **D_2%_** | 18.55 | 6.19 - 31.94 | 16.37 | 6.23 - 32.36 | 0.71 | 17.57 | 6.6 - 33.13 | **2.9** | 1 | --- | --- | --- |
|  | **D_0.5cm³_** | 18.7 | 6.53 - 30.67 | 17.09 | 6.55 - 31.0 | 0.56 | 17.96 | 6.97 - 32.13 | **4.6** | 1 | --- | --- | --- |
| **PBT** | **D_2%_** | 13.59 | 0.21 - 76.88 | 13.1 | 0.21 - 76.82 | 0.95 | 13.15 | 0.22 - 78.74 | 1.9 | 1 | --- | --- | --- |
|  | **D_0.5cm³_** | 14.78 | 0.22 - 79.53 | 14.68 | 0.22 - 80.02 | 1.64 | 14.7 | 0.22 - 81.61 | **2.1** | 1 | --- | --- | --- |
| **PTV** | **D_95%_** | 86.35 | 63.42 - 89.31 | 82.42 | 61.28 - 88.02 | -1.2 | 82.3 | 60.33 - 87.84 | -1.5 | **0.006** | **0.002** | **0.002** | **0.003** |
| **Liver Cohort** | | | | | | | | | | | | | |
| **spinal cord** | **D_2%_** | 6.05 | 0.3 - 18.32 | 5.32 | 0.3 - 15.65 | 1.08 | 5.86 | 0.3 - 18.05 | 1.0 | 1 | --- | --- | --- |
|  | **D_0.1cm³_** | 6.13 | 0.3 - 18.94 | 5.48 | 0.3 - 16.09 | 1.12 | 5.98 | 0.3 - 18.92 | 1.0 | 1 | --- | --- | --- |
| **esophagus** | **D_2%_** | 7.42 | 0.23 – 54.81 | 8.86 | 0.22 - 49.69 | 1.44 | 9.61 | 0.23 - 51.02 | **2.2** | 1 | --- | --- | --- |
|  | **D_0.5cm³_** | 7.42 | 0.23 – 54.81 | 8.83 | 0.21 - 48.79 | 1.43 | 9.68 | 0.22 - 48.21 | **2.3** | 1 | --- | --- | --- |
| **stomach** | **D_2%_** | 5.2 | 2.99 - 49.29 | 4.48 | 2.18 - 43.89 | **10.01** | 4.15 | 2.34 - 50.01 | **16.1** | 1 | --- | --- | --- |
|  | **D_0.5cm³_** | 5.84 | 3.52 - 62.73 | 5.07 | 2.97 - 72.31 | **10.52** | 5.03 | 3.26 - 100.58 | **38.8** | 1 | --- | --- | --- |
| **intestines** | **D_2%_** | 5.95 | 0.33 - 33.19 | 5.26 | 0.29 - 31.51 | -0.04 | 6.77 | 0.29 - 40.73 | **11.3** | 0.93 | --- | --- | --- |
|  | **D_0.5cm³_** | 19.49 | 0.47 - 61.87 | 12.97 | 0.35 - 75.22 | **13.35** | 15.71 | 0.4 - 96.52 | **34.7** | 1 | --- | --- | --- |
| **intestines partial** | **D_2%_** | 10.18 | 0.41 - 44.54 | 6.15 | 0.35 - 46.88 | **3.86** | 16.25 | 0.36 - 52.43 | **11.7** | 1 | --- | --- | --- |
|  | **D_0.5cm³_** | 18.76 | 0.46 - 61.87 | 18.82 | 0.38 - 72.53 | **10.66** | 22.79 | 0.39 - 96.52 | **34.5** | 1 | --- | --- | --- |
| **heart** | **D_2%_** | 3.93 | 0.18 - 27.83 | 5.96 | 0.18 - 23.18 | **4.38** | 7.77 | 0.18 - 26.25 | **3.8** | 1 | --- | --- | --- |
|  | **D_0.5cm³_** | 18.45 | 0.19 - 54.71 | 24.57 | 0.19 - 71.14 | **20.62** | 28.48 | 0.19 - 50.61 | **11.7** | 1 | --- | --- | --- |
| **kidney L** | **D_50%_** | 0.29 | 0.16 - 1.46 | 0.29 | 0.16 - 1.34 | 0.14 | 0.29 | 0.16 – 1.39 | 0.1 | 1 | --- | --- | --- |
| **kidney R** | **D_50%_** | 0.45 | 0.19 - 4.9 | 0.57 | 0.18 - 5.05 | 0.34 | 0.54 | 0.18 – 5.12 | 0.2 | 1 | --- | --- | --- |
| **liver** | **D_50%_** | 0.9 | 0.37 - 9.99 | 1.07 | 0.4 - 10.82 | 0.82 | 0.9 | 0.4 - 12.41 | **2.4** | 1 | --- | --- | --- |
| **PTV** | **D_95%_** | 87.38 | 58.45 - 136.87 | 82.09 | 52.36 - 110.89 | 0.68 | 75.10 | 50.71 – 90.99 | **-4.0** | **0.0448** | **0.0156** | **0.0078** | **0.0078** |

Baseline: initial plan on initial anatomy, DIR sum: dose accumulation via deformable image registration, DVH sum: dose accumulation via dose volume histogram summation, D_XX%_: dose to XX% of the volume, D_cm³_: dose to XX cm³ of the volume, ∆max: maximum individual difference to the baseline plan, p corr.: Bonferroni-Holm corrected p-value, p (DIR-BL): p-value for post-hoc comparison of DIR sum with the baseline plan, p (DVH-BL): p-value for post-hoc comparison of DVH sum with the baseline plan, p (DIR-DVH): p-value for post-hoc comparison of DIR sum with DVH sum.

**Supplementary Table 5. Deformable image registration quality measures.**

| **Lung** | | | | |
| --- | --- | --- | --- | --- |
|  | **Dice** |  | **Hausdorff Distance** | |
|  | Mean | SD | Mean | SD |
| **PBT** | 0.94 | 0.04 | 1.1 | 0.8 |
| **esophagus** | 0.89 | 0.07 | 1.3 | 1.0 |
| **heart** | 0.99 | 0.01 | 0.6 | 0.3 |
| **liver** | 0.97 | 0.01 | 1.6 | 0.8 |
| **lungs** | 0.97 | 0.01 | 2.9 | 0.8 |
| **spinal cord** | 0.91 | 0.03 | 1.3 | 1.2 |
| **Liver** | | | | |
|  | **Dice** |  | **Hausdorff Distance** | |
|  | Mean | SD | Mean | SD |
| **esophagus** | 0.74 | 0.12 | 6.0 | 3.8 |
| **heart** | 0.98 | 0.01 | 0.9 | 0.6 |
| **intestines** | 0.74 | 0.18 | 4.3 | 1.5 |
| **intestines partial** | 0.70 | 0.34 | 3.4 | 1.8 |
| **kidney L** | 0.97 | 0.01 | 0.6 | 0.3 |
| **kidney R** | 0.97 | 0.01 | 0.8 | 0.4 |
| **liver** | 0.96 | 0.01 | 2.6 | 1.7 |
| **spinal cord** | 0.90 | 0.04 | 1.5 | 1.3 |
| **stomach** | 0.88 | 0.12 | 2.1 | 1.4 |

**
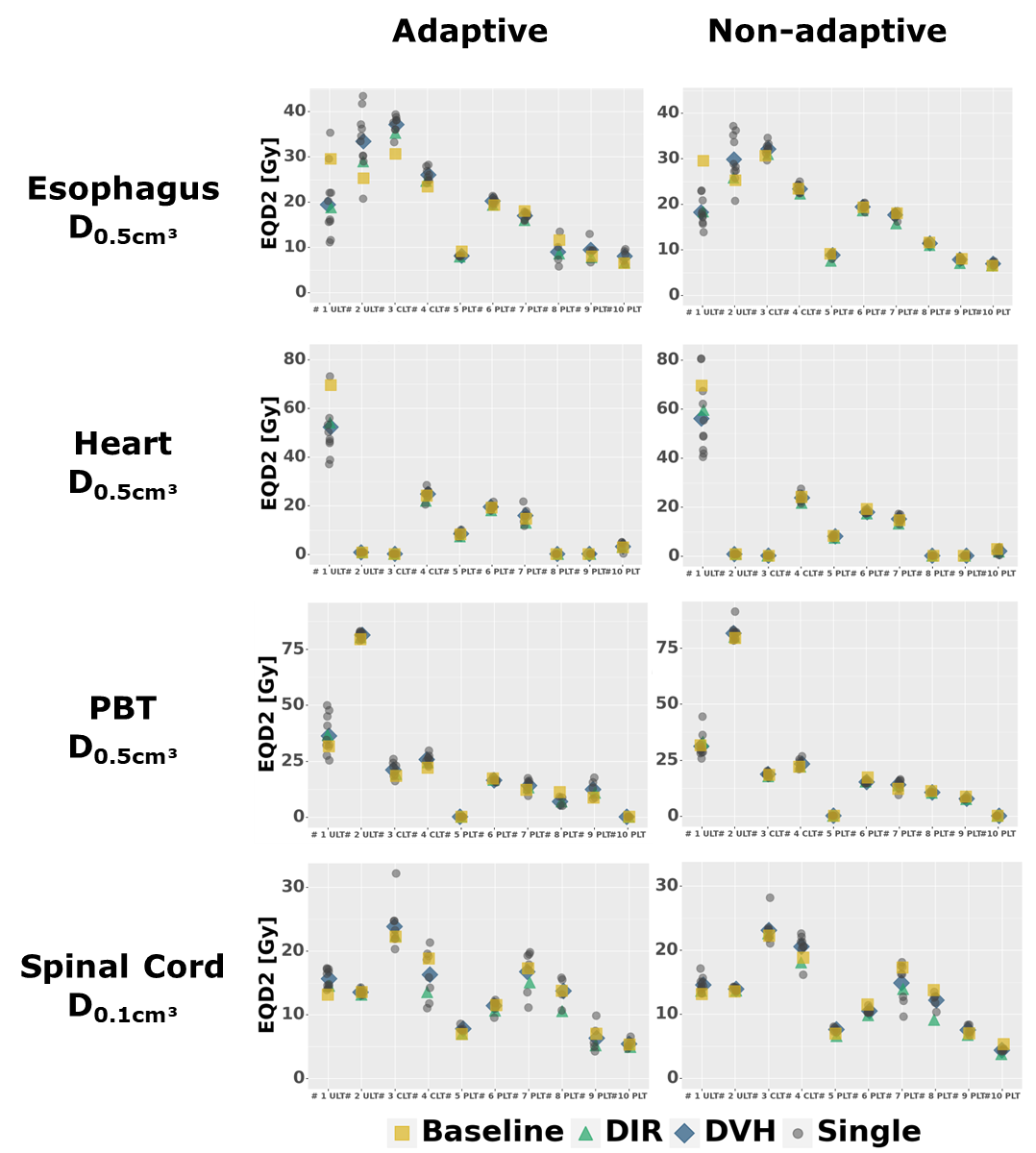
**

**Supplementary Figure 1. Dose Comparison in the lung cohort.** Comparison of near-maximum doses inside different organs-at-risk (OAR). **Adaptive** and **Non-Adaptive:** Dose comparison between baseline plans (yellow squares), deformable image registration-based dose accumulation (DIR, green triangles) and cumulative dose volume histograms (DVH, blue diamonds) on a single patient basis. Single doses (grey points) are plotted as well.

**
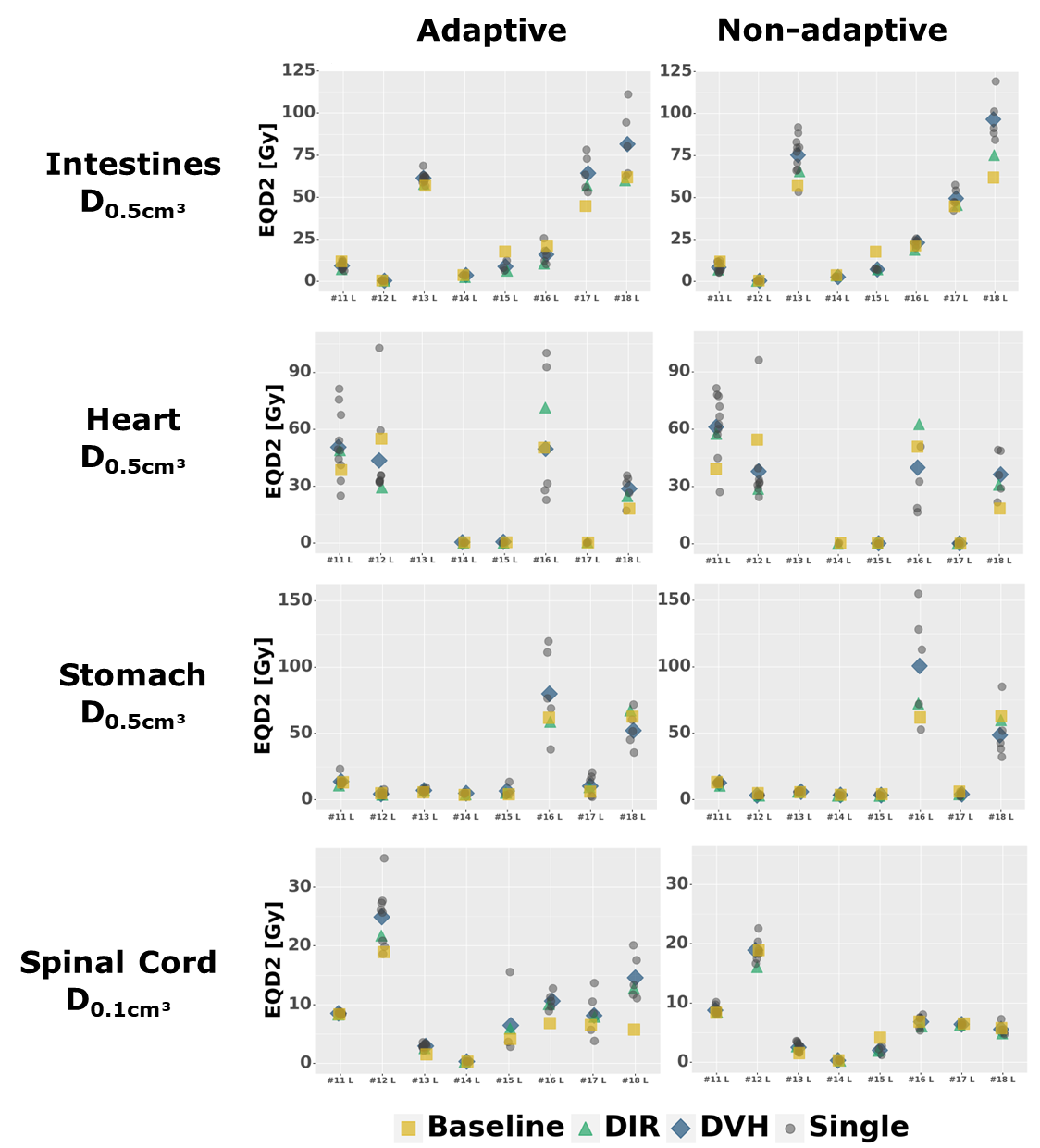
**

**Supplementary Figure 2. Dose Comparison in the liver cohort.** Comparison of near-maximum doses inside different organs-at-risk (OAR). **Adaptive** and **Non-Adaptive:** Dose comparison between baseline plans (yellow squares), deformable image registration-based dose accumulation (DIR, green triangles) and cumulative dose volume histograms (DVH, blue diamonds) on a single patient basis. Single doses (grey points) are plotted as well.

**
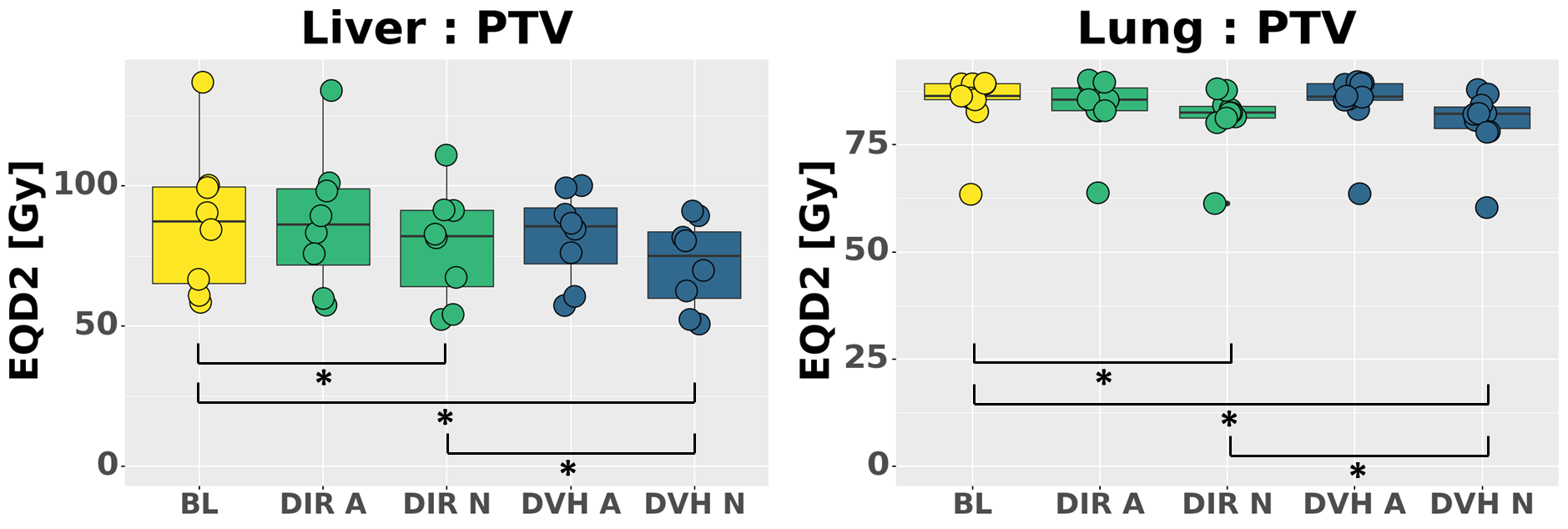
**

**Supplementary Figure 3. Planning Target Volume (PTV) doses.** Comparison of the dose to 95% of the PTV (D95%) between the baseline plan (BL), adaptive (A) and non-adaptive (N) treatment with Deformable Image Registration (DIR)-based dose accumulation and cumulative dose volume histograms (DVH). Doses were converted to the equivalent dose in 2 Gray (Gy) single doses (EQD2). *: statistically significant difference based on Friedman’s test with post-hoc pairwise Wilcoxon’s signed rank tests.

**Supplementary Figure 4. Deformable Image Registration (DIR) in the lung cohort.** PBT: proximal bronchial tree.


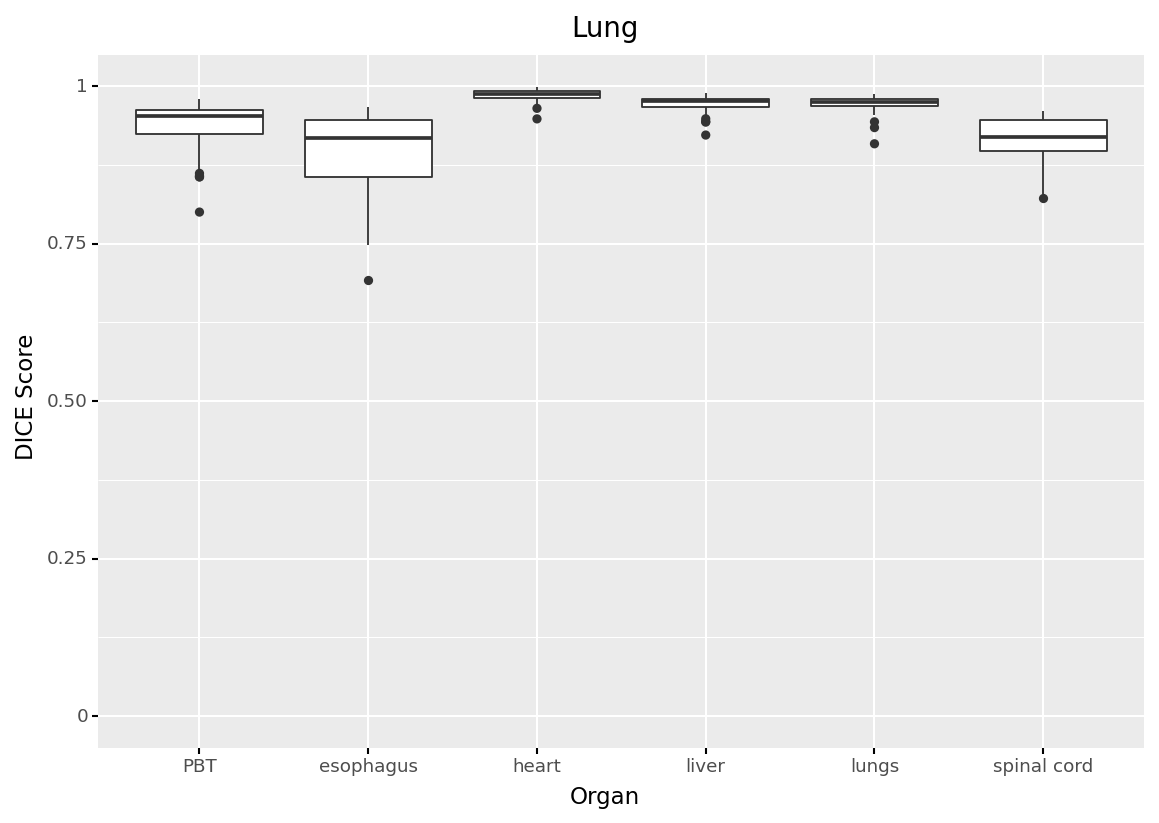

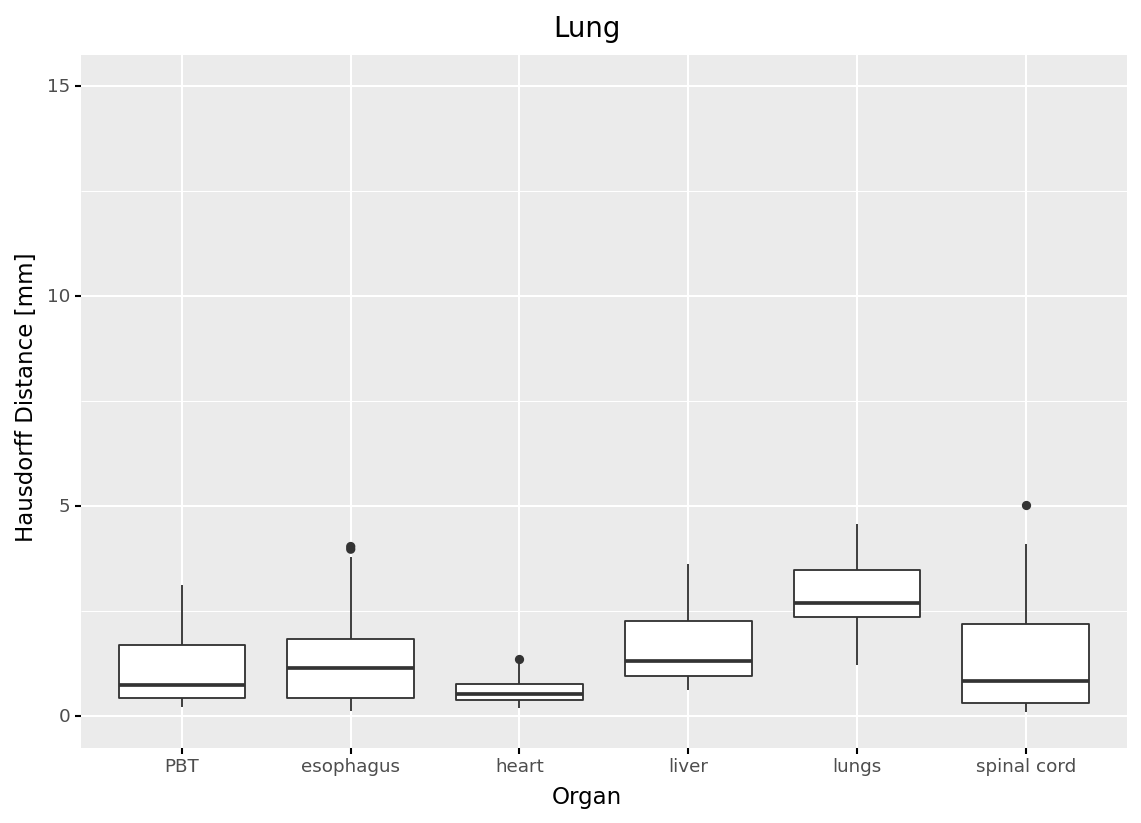


**Supplementary Figure 5. Deformable Image Registration (DIR) in the liver cohort.** Intestines part: intestinal subvolumes within 8 cm from planning target volume.


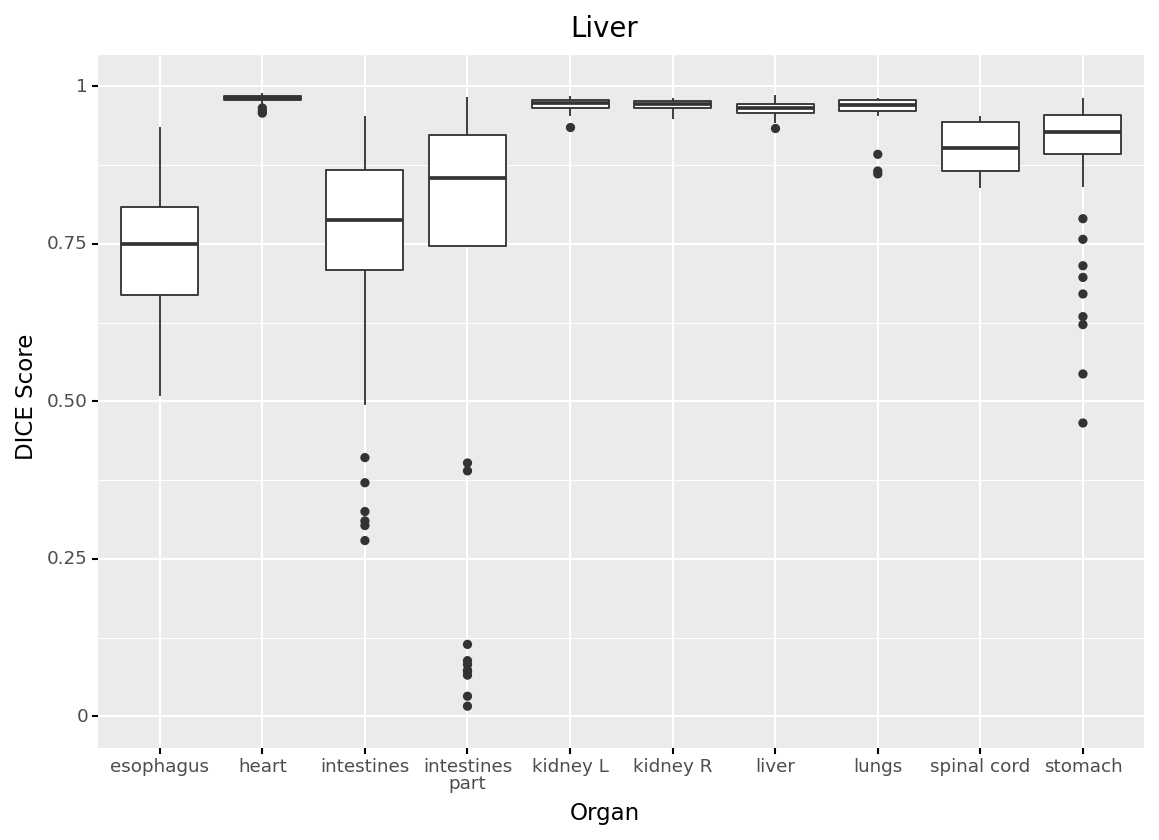

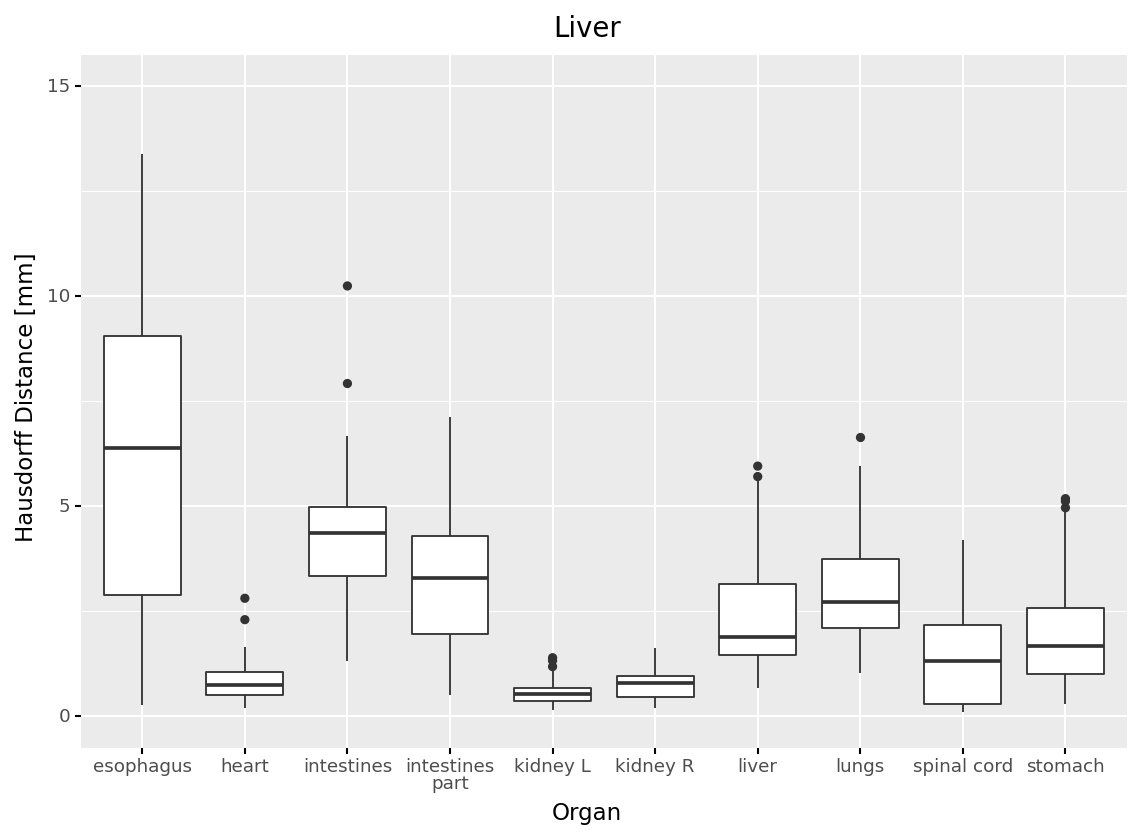

Supplement: Supplementary file 1 — Supplementary Material 1 [file 13014_2023_2284_MOESM1_ESM.docx]
